# Magnesium Restores Activity to Peripheral Blood Cells in a Patient With Functionally Impaired Interleukin-2-Inducible T Cell Kinase

**DOI:** 10.3389/fimmu.2019.02000

**Published:** 2019-08-27

**Authors:** Matthew K. Howe, Kennichi Dowdell, Amitava Roy, Julie E. Niemela, Wyndham Wilson, Joshua J. McElwee, Jason D. Hughes, Jeffrey I. Cohen

**Affiliations:** ^1^Laboratory of Infectious Diseases, National Institute of Allergy and Infectious Diseases, National Institutes of Health, Bethesda, MD, United States; ^2^Bioinformatics and Computational Biosciences Branch, National Institute of Allergy and Infectious Diseases, NIH, Hamilton, MT, United States; ^3^Department of Laboratory Medicine, Clinical Center, NIH, Bethesda, MD, United States; ^4^Lymphoid Malignancies Branch, Center for Cancer Research, National Cancer Institute, NIH, Bethesda, MD, United States; ^5^Merck Research Laboratories, Boston, MA, United States

**Keywords:** IL-2 inducible T cell kinase, ITK, Epstein-Barr virus, lymphomatoid granulomatosis, magnesium, immunodeficiency, T cell signaling

## Abstract

Interleukin-2-inducible T cell kinase (ITK) is critical for T cell signaling and cytotoxicity, and control of Epstein-Barr virus (EBV). We identified a patient with a novel homozygous missense mutation (D540N) in a highly conserved residue in the kinase domain of ITK who presented with EBV-positive lymphomatoid granulomatosis. She was treated with interferon and chemotherapy and her disease went into remission; however, she has persistent elevation of EBV DNA in the blood, low CD4 T cells, low NK cells, and nearly absent iNKT cells. Molecular modeling predicts that the mutation increases the flexibility of the ITK kinase domain impairing phosphorylation of the protein. Stimulation of her T cells resulted in reduced phosphorylation of ITK, PLCγ, and PKC. The CD8 T cells were moderately impaired for cytotoxicity and degranulation. Importantly, addition of magnesium to her CD8 T cells *in vitro* restored cytotoxicity and degranulation to levels similar to controls. Supplemental magnesium in patients with mutations in another protein important for T cell signaling, MAGT1, was reported to restore EBV-specific cytotoxicity. Our findings highlight the critical role of ITK for T cell activation and suggest the potential for supplemental magnesium to treat patients with ITK deficiency.

## Introduction

Interleukin-2-inducible T cell kinase (ITK) is expressed in T cells, NK cells, invariant NKT (iNKT) cells, and mast cells (1). ITK is a component of the signaling pathway that leads to T cell activation, where it serves to amplify T cell receptor (TCR) signaling. Additionally, ITK is also involved in T cell proliferation, differentiation, and cytotoxic activity (2, 3). Patients with mutations in ITK have low numbers of iNKT cells and impaired calcium flux in T cells after TCR stimulation. ITK is critical for control of infection with Epstein-Barr virus (EBV) and several patients with mutations in ITK have been reported with markedly elevated levels of EBV in the blood and B lymphoproliferative disease. Some patients have had EBV-positive Hodgkin lymphoma, large B cell lymphoma, or lymphomatoid granulomatosis. The largest review of patients with mutations in ITK showed that 6 died [one after hematopoietic stem cell transplant (HSCT)], one was alive after HSCT, and one was in remission after chemotherapy (4). Thus, improved therapies are needed for ITK deficiency.

Here we report a novel homozygous missense in the kinase domain of ITK in a patient that was diagnosed with EBV-positive lymphomatoid granulomatosis. This mutation caused a functional defect in ITK, resulting in impaired calcium flux in T cells, reduced phosphorylation of PLCγ and other downstream signaling molecules, and reduced cytotoxic activity. We found that *in vitro* magnesium supplementation restored cytotoxic activity of the patient's cells. This suggests that supplemental magnesium has potential as a new therapeutic approach for patients with EBV-lymphoproliferative disease due to ITK deficiency.

## Methods

### Cell Culture

Peripheral blood mononuclear cells (PBMCs), resuspended in RPMI with glutaxmax (Gibco), supplemented with 10% fetal bovine serum (FBS, Lonza), were expanded with anti-CD3/CD28 (Biolegend) antibodies for 48 h, followed by addition of 100 U/mL of IL-2. Cells were maintained by the addition of 100 U/mL of IL-2 every 24 h. Cells that were maintained in IL-2 were rested overnight in RPMI and 2% FBS without Glutamax or IL-2 prior to stimulation with anti-CD3 antibodies. The mean percent cell death for CD8 cells in culture when the media was changed from 10% FBS with IL-2 to 2% FBS without IL-2 over night was similar between the patient (0.89%, mean of 5 replicates) and four controls (0.83%, mean of 5 replicates). RPMI contains 0.407 mM magnesium sulfate, and where indicated, cells were cultured for 5 days in supplemental magnesium sulfate at a concentration of 1 mM (unless otherwise specified) that was added to RPMI with Glutamax and 10% FBS.

### Flow Cytometry

Cells were rested overnight in RPMI and 2% FBS without Glutamax or IL-2. Cells were then labeled with PerCP anti-CD8 (Life Technologies) and APC-Cy7 anti-CD3 antibodies, prior to stimulation with anti-CD3 antibodies for the indicated times. Where indicated, cells were cultured for 5 days in supplemental magnesium sulfate, as described above. BD Cytofix/Cytoperm™ fixation and permeabilization solution was then added to cells. The cells were then washed in BD Perm/Wash™ buffer followed by incubation with antibody. Cells were washed twice with BD Perm/Wash™ buffer and analyzed on a BD FACS CANTO II. Cells were identified that were CD8 positive and the MFI was determined.

### DNA Sequencing and Whole Exome Analysis

Sequencing was performed as previously described (5). Briefly, genomic DNA was isolated from the patient, and DNA sequencing was performing using a SureSelect Human All Exon 50 Mb kit (Agilent Technologies) along with sequencing by Illumina HiSeq sequencing. Mutations were confirmed by Sanger sequencing.

Whole-exome analysis was performed as previously described (5). Briefly, DNA reads were mapped to hg19 human genome reference by Burrows-Wheeler Aligner. Single nucleotide variant, insertion, and deletion calling was performed with the Genome Analysis Toolkit (Broad Institute) and annotated using an in-house custom analysis pipeline to filter and prioritize variants.

### Immunoblots

SDS-PAGE was performed and after transfer to nitrocellulose membranes, blots were incubated with primary antibodies overnight at 4°C. The next day, membranes were washed three times in Tris-buffered saline with 0.01% Tween 20 (TBS-T), incubated in horseradish peroxidase conjugated secondary antibody for 1 h, washed three times in TBS-T, and incubated with SuperSignal™ West Pico Chemiluminescent Substrate (ThermoFisher).

### Antibodies

Anti-ITK, anti-PLCγ, anti-pPLCγ Tyr783, anti-pPKC βII Ser660, anti-GAPDH, and anti-actin antibody, and the corresponding secondary antibodies were purchased from Cell Signaling. Anti-pITK Tyr 512 antibody was purchased from BD Bioscience. PE anti-pITK Tyr512 was purchased from eBioscience.

### Molecular Dynamic (MD) Modeling

MD simulations were done using CHARMM-c39 (6) and run with the software ACEMD (7). All structures were solvated in a TIP3 water box with Na^+^ and Cl^−^ added as counter ions to reach an ionic strength of 0.15 M. Following an initial equilibration, five 0.5 μs of MD simulations each of WT and mutant ITK were performed at constant temperature and pressure. Collectively, 5 simulations produced 2.5 μs of trajectories each for WT and mutant ITK. Root mean square fluctuation (RMSF), which measures the deviation of the atoms in a residue from their mean positions during the simulation, was determined. The RMSF analyses (performed using 95% confidence intervals) and the plots (shown with 80% confidence intervals) were determined by the bootstrapping method.

### Calcium Flux Assay

PBMCs maintained in IL-2 were rested overnight in RPMI with 5% FBS. Cells were washed once in wash buffer (120 mM NaCl, 20 mM HEPES, 4.7 mM KCl, 1.2 mM KH_2_PO_4_, 1.2 mM MgSO_4_, 1.2 mM CaCl_2_ and glucose) and incubated with 2 μM Indo-1 (Invitrogen) for 30 min. Cells were washed twice in wash buffer and incubated on ice until analysis on a BD LSR II. Cells were first analyzed for 30 s to establish a baseline, next 10 μg/ml of anti-CD3 was added. After an additional 30 s, CD3 was cross-linked by the addition of 10 μg/ml IgG (Biolegend) for 4 min. Finally, 1 μM Calcimycin (Invitrogen) was added as a positive control for calcium flux for 1 min. Calcium flux was graphed as a function of the level of Indo-1 (Fluo-4) over time using the kinetics function on FlowJo software (FlowJo LLC).

### Cytotoxicity Assay

The cytotoxicity assay was performed as previously described (8). Briefly, L1210 lymphocytic leukemia murine cells (American Type Culture Collection) were grown in RPMI, Glutamax and 10% FBS. Prior to incubation with PBMCs, L1210 cells were labeled with TFL4-APC. Expanded PBMCs were maintained in RPMI with Glutaxmax, supplemented with 10% FBS, and 100 U/mL of IL-2 for 48 h before enrichment of CD8 cells by negative selection (Stem Cell) per the manufacturer's instructions. Enriched CD8 cells and L1210 cells were then co-cultured at the indicated ratios for 4 h, in RPMI, 10% FBS, 20 U/mL IL-2, 2.5 μg anti-human CD3 antibody (2 μg/mL) which is required to activate the T cells, and anti-human Fas (Millipore) to block the Fas pathway and ensure Fas-independent killing. BD Cytofix/Cytoperm™ fixation and permeabilization solution was then added to cells. The cells were then washed in BD Perm/Wash™ buffer followed by incubation with PE-anti-caspase3 antibody (BD Bioscience). Cells were washed twice with BD Perm/Wash™ buffer and analyzed on a BD FACS CANTO II. L1210 cells were identified as APC-positive and cytotoxicity was determined based on L1210 cells that were positive for caspase-3.

### CD107a Degranulation

PBMCs were rested overnight in RPMI, 2% FBS, and 10 U/mL IL-2. The next day cells were rested for 1 h in media without IL-2. Cells were incubated with an APC anti-CD107a antibody (Biolegend) and were stimulated with either anti-CD3/CD28 Dynabeads (Gibco) or PMA and ionomycin. After 2 h cells were placed on ice, washed once in ice cold PBS, labeled with PerCP anti-CD8 and BV421 anti-CD3 antibodies (BD) antibodies, and analyzed on a BD FACS CANTO II. For analysis, cells were gated on CD3 and CD8, followed by CD107a, which indicates that cells that are degranulating, and analyzed on a BD FACS CANTO II.

### Statistical Analyses

Statistical analysis (*T*-test or 2way ANOVA) was performed using Prism (Graphpad) software. *P*-values < 0.05 were considered statistically significant.

### Study Approval

This study was carried out in accordance with the recommendations of the Institutional Review Boards (IRBs) of the National Institute of Allergy and Infectious Diseases (NIAID) and the National Cancer Institute (NCI) with written informed consent from all subjects. All subjects gave written informed consent in accordance with the Declaration of Helsinki. The protocols were approved by the NIAID and NCI IRB (ClinicalTrials.gov identifiers NCT00001379 and NCT01011712). Healthy blood bank donors signed informed consents on a protocol approved by the Warren G. Magnuson Clinical Center of the National Institute of Health.

## Background

### Case Presentation

The patient is a 32-year-old Jordanian female who presented with lung nodules at age 22 and biopsy showed grade 1 lymphomatoid granulomatosis. Three months later she developed respiratory failure and was treated with corticosteroids and cyclophosphamide, but the lung nodules increased in size. A biopsy showed grade 3 lymphomatoid granulomatosis with EBV-positive B cells. She was treated with interferon-α without a good response, and received six cycles of EPOCH-R (etoposide, prednisone, vincristine, cyclophosphamide, doxorubicin, rituximab) chemotherapy (Table 1). She relapsed, was treated with interferon-α, relapsed again, was retreated with again with six cycles of EPOCH-R chemotherapy, and is currently in remission. All samples were analyzed when the patient was between 26 and 31 years of age, and all were at least 21 months after her last chemotherapy. Her most recent cell counts show reduced percentage of CD4 cells (22.1%, normal 31.9–62.2%), NK cells (3.1%, normal 6.2–34.6%), and iNKT cells (0.028%, normal <0.1%), and reduced numbers of NK cells (56/ul, normal 126–729/ul) (Table S1). She has a reduced percentage of naive CD4 cells (CD62L^+^ CD45RA^+^ 2.9%, normal 7.6–37.75%), and increased percentages of effector memory CD8 cells (CD62L^−^ CD45RA^−^ 9.6%, normal 1.1–9.2%), and terminally differentiated effector memory CD8 cells (CD62L^−^ CD45RA^+^ 15.2%, normal 0.7–7.8%). Her CD4, CD8, and CD19 cell numbers are normal as is her IgG level. Prior CD4 counts over the last 8 years were all low ranging from 92–314/ul (normal 359–1,565/ul) and NK cell counts were also low ranging from 27–73/ul (normal 126–729/ul). Her blood EBV DNA PCR is elevated at 38,000 copies/ml (normal <200 copies/ml) and has been persistently elevated. The patient has no history of severe infections, but had hemolytic anemia in childhood. She has two brothers who are healthy and her parents are first cousins.

**Table 1:** Clinical diagnosis and treatment of the patient.

**Date**	**Clinical diagnosis**	**Treatment**
12/2018	LyG grade 1, lung nodules	Corticosteroids, cyclophosphamide
2/2009	LyG grade 3, lung nodules	IFN-α
2/2009 to 6/2009	LyG grade 3, lung nodules	EPOCH-R x 6 cycles
2/2010 to 9/2010	Relapsed LyG, lung nodules	IFN-α
9/2010 to 1/2011	Progressive disease, lung nodules and spleen masses	EPOCH-R x 6 cycles
3/2011 to present	Remission	none

*LyG, lymphomatoid granulomatosis; EPOCH-R, etoposide, prednisone, vincristine, cyclophosphamide, doxorubicin, rituximab*.

## Results and Discussion

**Figure 1:**
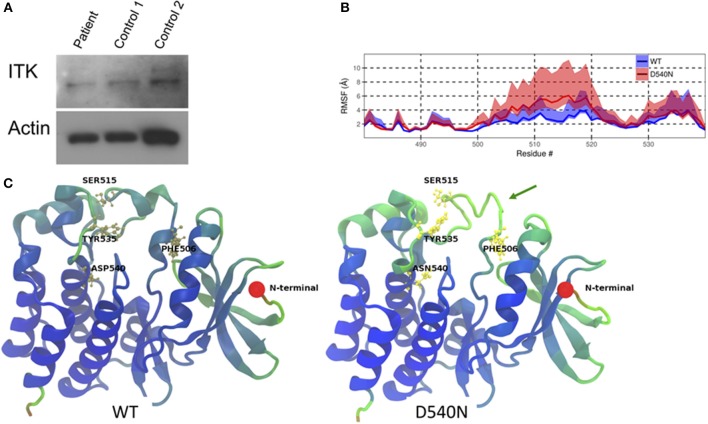
ITK mutation in the patient. **(A)** ITK protein is present in the patient's peripheral blood mononuclear cells at a similar level to control. Actin is a protein loading control. **(B)** The root mean square fluctuation (RMSF) for wild-type ITK (WT) and the mutant ITK in the patient (D540N) is shown with the 80% confidence interval highlighted. **(C)** Average structure from the simulations, displaying the increased flexibility of the ITK kinase domain, which causes the alpha helical turns in residues 504–506 and residues 510 to 513 to be missing from the mutant compared to the WT ITK, indicated by the green arrow. The residues are colored according to their RMSF values, with blue representing low-, green representing the mid- and red representing the high-RMSF values.

Whole exome sequencing from peripheral blood mononuclear cells of the patient showed a novel homozygous missense mutation in the kinase domain of ITK (*ITK* NM_005546 c.1618G>A p.D540N). This mutation resulted in an amino acid change from aspartic acid to asparagine at residue 540, which is a highly conserved residue. The patient comes from a consanguineous family and has two healthy brothers. One brother was confirmed to be heterozygous for the same mutation in ITK. Immunoblotting showed similar levels of ITK in the patient cells compared to cells from two healthy controls (Figure 1A).

Through molecular dynamic modeling we sought to determine if the mutation might cause a conformational change in ITK. The crystal structure of the kinase domain of ITK has been reported previously (9) and WT ITK (PDBid:1SNU) was used as a starting model for the molecular dynamic (MD) simulation. Flexibility of the protein was calculated using the root mean square fluctuation (RMSF), which measures the deviation of the atoms in a residue from their mean positions during the simulation. The mutant ITK showed increased flexibility, as determined by an increase in RMSF, at amino acids 506-520, particularly for residues F506 and S515 compared with WT ITK (Figure 1B). Due to the increase in flexibility in residues 506–520 in the ITK mutant, the alpha helical turn at residues 504–506 and 510–513 in WT ITK is absent in the mutant ITK (Figure 1C). The increased flexibility in the kinase domain caused by the mutation, predicts that residue Y512 is in a conformation that is less amenable to phosphorylation resulting in reduced activation of ITK.

**Figure 2:**
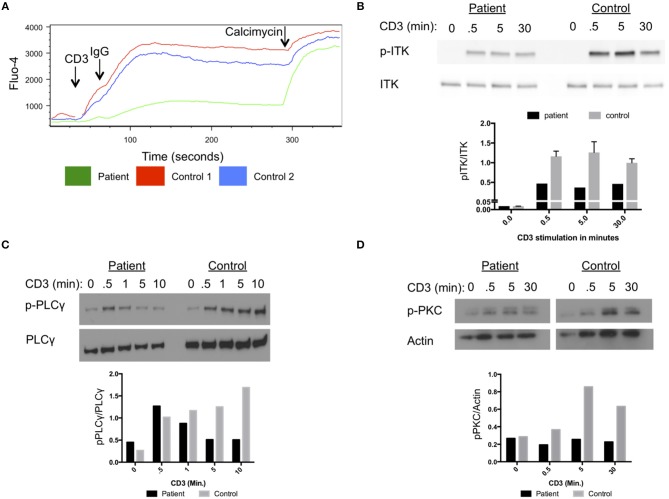
Impaired T cell receptor signaling in cells from the patient with homozygous ITK mutations. **(A)** Impaired calcium flux in the patient compared to healthy controls following anti-CD3 crosslinking, plotted by Fluo-4 as a function of time. **(B)** Impaired phosphorylation of ITK in patient cells following stimulation for the indicated times with anti-CD3 antibody, compared to control cells. **(C)** Phosphorylation of PLCγ at various times after CD3 stimulation in patient and control. Total PLCγ served as a loading control. **(D)** Phosphorylation of PKC in patient cells following CD3 stimulation at various times compared to control. Actin served as a loading control. **(B–D)** The graphs show quantification of the blots. The experiments were repeated three times and a representative result is shown.

Our patient had impaired calcium flux in lymphocytes cells after stimulation with anti-CD3 antibodies and cross-linking with IgG (Figure 2A). Phosphorylation of ITK at amino acid Y512 in the patient cells was reduced following stimulation with anti-CD3 antibodies compared to a healthy control (Figure 2B). Activated ITK induces phosphorylation of PLC-γ, and PKC. Phosphorylation of PLC-γ and PKC were reduced in the patient's cells compared to healthy control cells (Figures 2C,D). Thus, the D540N mutation in ITK resulted in a functionally impaired protein, with reduced phosphorylation of ITK and its downstream targets PLC-γ and PKC following stimulation through the TCR.

**Figure 3:**
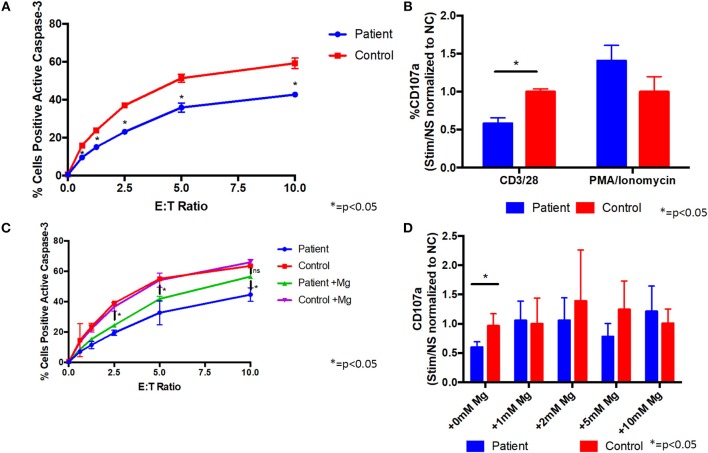
Supplemental magnesium restores impaired degranulation and cytotoxicity associated with T cell signaling *in vitro* in cells from a patient with homozygous ITK mutations. **(A)** Cytotoxicity is impaired in CD8+ cells from the patient compared to CD8+ cells from a healthy control. Patient or healthy control CD8+ cells were incubated with L1210 murine cells with anti-CD3 and anti-Fas antibodies. Cytotoxicity was measured by flow cytometry as the percentage of L1210 cells positive for active caspase-3. E:T ratio is the effector cell to target cell ratio. Data represent mean ± standard deviation (SD). **(B)** Degranulation is impaired in CD8+ cells from the patient following CD3/CD28 stimulation. Prior to stimulation with anti-CD3/CD28 antibodies, anti-CD107a antibody (a marker of degranulation) was added to cells from the patient or healthy normal control (NC). Following stimulation, patient and control cells positive for CD107a were measured by flow cytometry. Degranulation was quantified as the fold change of the percent of CD107a positive cells from the stimulated (Stim) to non-stimulated (NS) samples and normalized to the healthy control. PMA/ionomycin was used as a positive control, which shows similar degranulation in patient and control cells. Data represent mean ± SD. **(C)** Cytotoxicity in CD8+ cells following culture with 1 mM supplemental magnesium (Mg) or no additional magnesium. Data represent mean ± SEM. **(D)** Degranulation when patient and healthy control cells were cultured in supplemental Mg for 5 days. Data represent mean ± SD. **(A–D)** Asterisk indicates *p* < 0.05. anti-CD3, anti-CD28, and anti-CD107a antibodies were from Biolegend, PE-anti-caspase 3 antibody was from BD Bioscience, and anti-Fas antibody was from Millipore. All experiments were repeated two to four times and pooled data are shown.

Since the patient's T cells showed impaired activation after TCR stimulation, we evaluated their cytotoxic activity. Co-culture of the patient's CD8 cells with L1210 cells resulted in reduced caspase-3 activation of the L1210 cells, indicative of impaired cytotoxicity, compared with control cells (Figure 3A). We also found that the patient's CD8 cells were impaired for degranulation, as measured by reduced CD107a on the cell membrane, following stimulation with anti-CD3/CD28 antibodies, compared to control cells (Figure 3B). In addition, stimulation of the patient's CD8 cells with PMA/ionomycin showed little difference with control cells (Figure 3B), indicating that the patient's cells can be sufficiently activated, when the proximal TCR pathway is bypassed. Taken together, these results indicate that the patient's CD8 cells are impaired for cytotoxic activity through the TCR pathway.

Like ITK, magnesium transporter 1 (MAGT1) is critical for T cell signaling, and patients with mutations in MAGT1 have high levels of EBV, reduced T cell activation, with reduced calcium flux and phosphorylation of PLCγ and PKC, and impaired cytotoxicity and degranulation (10, 11). Treatment of cells from patients with mutations in MAGT1 with supplemental magnesium partially restores the defects in T cell cytotoxicity. Due to the similarities in clinical phenotypes and in the defects in T cell signaling pathways in patients with ITK and MAGT1, we determined if magnesium supplementation might rescue some of the defects observed in our patient's cells. Supplemental magnesium, at a similar concentration and duration used in MAGT1 for *in vitro* experiments (10), partially restored the cytotoxicity defect in cells from the ITK patient (Figure 3C). Treatment of the patient's cells with supplemental magnesium rescued the defect in degranulation, such that there was no longer a significant difference between the patient's cells and control cells (Figure 3D and Figure S1). These data indicate that supplemental magnesium can rescue some of the defects observed in T cells from the ITK patient and has a potential for treatment. This is consistent with previous results observed in patients with XMEN disease, where supplemental magnesium also restored cytotoxicity defects (10, 11). The patient declined a trial of oral magnesium supplementation to determine if it would improve the cytotoxicity of her T cells and reduce her EBV viral load.

## Concluding Remarks

Here we report a novel homozygous missense mutation in the kinase domain of ITK in a patient with EBV-positive lymphomatoid granulomatosis and show that defects in cytotoxicity and degranulation activity of the patient's cells can be rescued with supplemental magnesium *in vitro*. This represents the twentieth patient identified with a mutation in ITK (4, 12–21). Our patient shares many characteristics with these prior patients including low numbers of CD4 and iNKT cells, and EBV-positive pulmonary disease. Our patient is from Jordan; most of the other patients with homozygous mutations are also from the Middle East. Our patient presented at 22 years old, older than all of the other patients who presented at a mean of age of 5.8 years, ranging from 0.3 to 18 years at presentation. Fifteen of the previously reported patients with ITK mutations died from their disease or required hematopoietic stem cell transplantation, while 4 patients went into remission after chemotherapy, and the status of one patient is unknown. Our data predicts that the D540N mutation in ITK causes a conformational change in the kinase domain of the protein, which could reduce its phosphorylation and its ability to activate downstream targets and coordinate T cell cytotoxicity. Molecular modeling predicts that the increased flexibility of residues 506 to 520 could cause residue Y512 to be in a conformation that is less accessible to phosphorylation. This was supported by the reduced phosphorylation of ITK at residue Y512 and the impaired activation of ITK downstream targets, PLCγ and PKC, and reduced cytotoxicity.

We found that magnesium supplementation restored the cytotoxic and degranulation defects observed in our patient's cells. Oral magnesium supplementation has also been shown to improve cytotoxicity in T cells and reduce the fraction of EBV-positive cells in the blood in patients with mutations in MAGT1 (10). ITK has been reported to act as a magnesium sensor (22); therefore magnesium supplementation might partially complement the role of ITK in degranulation and cytotoxicity. In addition, impaired ITK activation was reported to be the primary reason for impaired T cell receptor activation in patients with XMEN disease; an influx of magnesium reconstituted ITK activity in cells from patients with XMEN disease (23). Alternatively, magnesium is known to bind to several protein kinases and supplemental magnesium may indirectly enhance T cell receptor signaling (24). Further experiments will be needed to determine if these, or other activities of magnesium are responsible for restoring degranulation and cytotoxicity *in vitro* in the patient's cells.

The observation that supplemental magnesium *in vitro* restored T cell cytotoxicity in cells from patients with mutations in ITK and MAGT1, and the reduction in the percentage of EBV-positive B cells in the blood of patients with mutations in MAGT1 given oral magnesium, suggests that oral magnesium might also be effective for patients with mutations in ITK. However, the specific ITK mutation may influence the efficacy of oral magnesium. Future studies may show that such treatment might delay or reduce the need for hematopoietic stem cell transplantation in these patients.

## Ethics Statement

The patient signed informed consent on protocols approved by the Institutional Review Boards of the National Institute of Allergy and Infectious Diseases and the National Cancer Institute. Healthy blood bank donors signed informed consents on a protocol approved by the Warren G. Magnuson Clinical Center of the National Institute of Health.

## Author Contributions

MH and KD performed the research. WW and JC provided patient care. AR performed molecular modeling. JN, JM, and JH performed exome and Sanger sequencing. MH performed statistical analyses. MH, KD, and JC designed the experiments and analyzed the data. MH and JC wrote the paper.

### Conflict of Interest Statement

JM and JH were employed by Merck Research Laboratories at the time of the work. The remaining authors declare that the research was conducted in the absence of any commercial or financial relationships that could be construed as a potential conflict of interest.
