# Supplementary material for: Magnesium Restores Activity to Peripheral Blood Cells in a Patient With Functionally Impaired Interleukin-2-Inducible T Cell Kinase
Source: Front Immunol. 2019 Aug 27;10:2000. doi: 10.3389/fimmu.2019.02000 (PMC6718476; doi:10.3389/fimmu.2019.02000)
Supplement: Supplementary file 1 [file Table_1.DOCX]

**Supplemental Table**

**Supplemental Table 1. Lymphocyte phenotypes of patient (age 31)**

| Cell Type | Patient (%) | Healthy Controls (%) |
| --- | --- | --- |
| CD3 | 1412/uL (78.0) | 714-2266/uL (60.0-83.7) |
| CD4 | 400/uL (**22.1**) | 359-1565/uL (31.9-62.2) |
| CD8 | 776/uL (42.9) | 178-853/uL (11.2-34.8) |
| CD19 | 304/uL (16.8) | 61-321/uL (3.3-19.3) |
| NK | 56/uL (3.1) | 126-729/uL (6.2-34.6) |
| Naive CD4 | **52** (**2.9**) | 102-1041 (7.6-37.7) |
| Central memory CD4 | 293 (16.2) | 162-614 (10.4-30.7) |
| Effector memory CD4 | 168 (9.3) | 42-225 (2.3-15.6) |
| Terminally differentiated memory CD4 | 13 (0.7) | 0-29 (0-1.5) |
| Naive CD8 | 174 (9.6) | 85-568 (5.7-19.7) |
| Central memory CD8 | 87 (4.8) | 25-180 (1.5-10.3) |
| Terminally differentiated memory CD8 | 174 (9.6) | 24-175 (1.1-9.2) |
| Naive CD8 | 275 (15.2) | 11-172 (0.7-7.8) |

Naïve CD4 (CD62L^+^,CD45RA^+^), central memory CD4 (CD62L^+^,CD45RA-), effector memory CD4 (CD62L^-^, CD45RA^-^), terminally differentiated effector memory CD4 (CD62L^-^, CD45RA^+^), naïve CD8 (CD62L^+^, CD45RA^+^), central memory CD8 (CD62L^+^, CD45RA^-^), effector memory CD8 (CD62L^-^, CD45RA^-^), terminally differentiated effector memory CD8 (CD62L^-^, CD45RA^+^) 11-172. Bold=below limit of normal, Underline=above upper limit of normal, ND=not done.
