# Supplementary figures and images for: Magnesium Restores Activity to Peripheral Blood Cells in a Patient With Functionally Impaired Interleukin-2-Inducible T Cell Kinase
Source: Front Immunol. 2019 Aug 27;10:2000. doi: 10.3389/fimmu.2019.02000 (PMC6718476; doi:10.3389/fimmu.2019.02000)

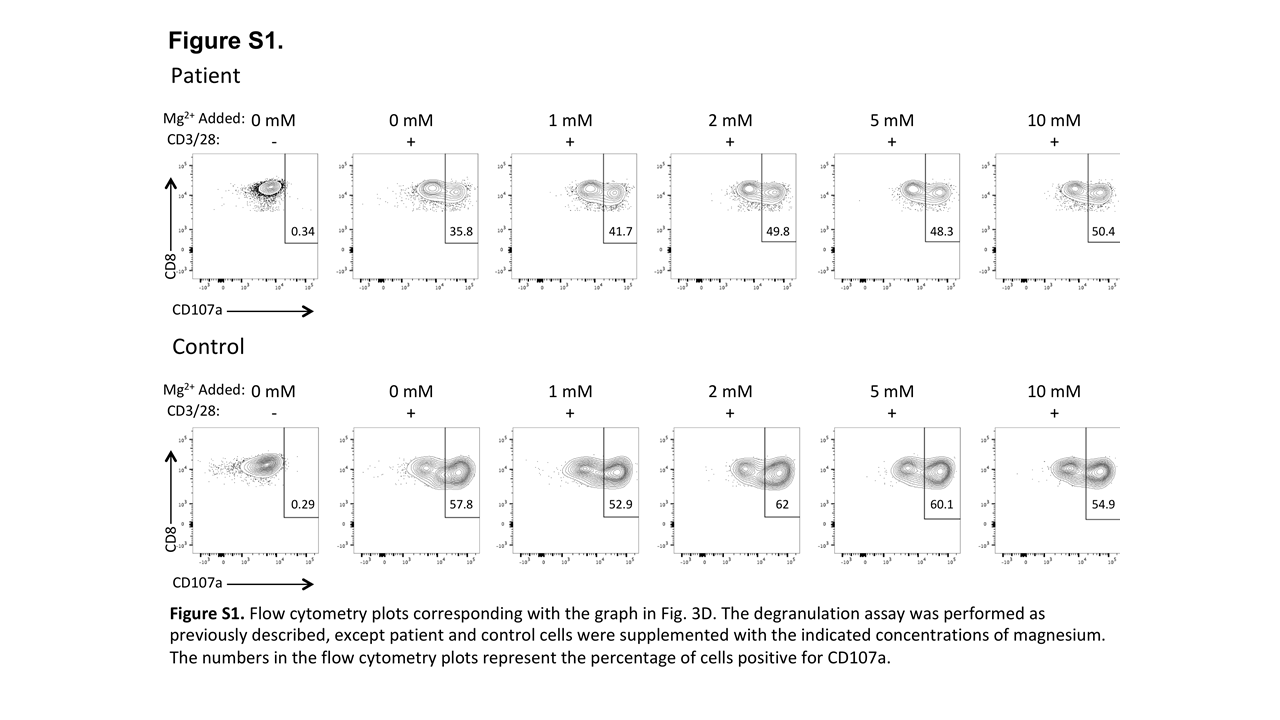

Supplement: Supplementary file 2 [file Image_1.TIF]
